# Comparative Outcomes of 1-Level vs. 2-Level Anterior Cervical Discectomy and Fusion: A Systematic Review and Meta-Analysis

**DOI:** 10.3390/jcm14196788

**Published:** 2025-09-25

**Authors:** Joseph E. Nassar, Ashley Knebel, Manjot Singh, Michael J. Farias, Nicolas L. Carayannopoulos, Zvipo M. Chisango, Negin Fani, Mohammad Daher, Eren O. Kuris, Bassel G. Diebo, Alan H. Daniels

**Affiliations:** Department of Orthopaedic Surgery, Brown University, Providence, RI 02914, USA; jen06@mail.aub.edu (J.E.N.); ashley_knebel@brown.edu (A.K.); manjot_singh@alumni.brown.edu (M.S.); michael_farias@brown.edu (M.J.F.); nlc90@rwjms.rutgers.edu (N.L.C.); zvipo.chisango@emory.edu (Z.M.C.); neginfani@gmail.com (N.F.); mohdaher06@gmail.com (M.D.); eren_kuris@brown.edu (E.O.K.); dr.basseldiebo@gmail.com (B.G.D.)

**Keywords:** anterior cervical discectomy and fusion, 1-level, 2-level, dysphagia, patient-reported outcome measures

## Abstract

**Background/Objectives**: Cervical spine disease requiring surgical intervention is a major cause of disability. Anterior cervical discectomy and fusion (ACDF) is a well-established procedure for treating cervical pathology; however, there remains no consensus on whether 1-level versus 2-level ACDF yields comparable outcomes. This study compares 1-level versus 2-level ACDF by evaluating surgery-related and postoperative outcomes, radiographic parameters, and patient-reported outcome measures (PROMs). **Methods**: PubMed, Embase, Scopus, and Cochrane Library were searched through 10 July 2024. Studies comparing 1-level with 2-level ACDF were included. Data on operating room (OR) time, estimated blood loss (EBL), length of hospital stay (LOS), complications, and PROMs, including Neck Disability Index (NDI) and Visual Analogue Scale (VAS) for neck and arm pain, were extracted. **Results**: Thirteen studies met our inclusion criteria, comprising 2091 patients (1078 undergoing 1-level and 1013 2-level ACDF). No statistically significant differences were observed in EBL or LOS between the cohorts. However, the 2-level ACDF group showed significantly longer OR times (*p*-value < 0.001) and higher odds of developing dysphagia (*p*-value = 0.05). Patients undergoing 2-level ACDF showed greater correction in cervical lordosis. Both cohorts reported similar statistically and clinically significant improvements in VAS neck and NDI scores at final follow-up. There was no difference in adjacent segment disease or revision surgery at final follow-up. **Conclusions**: Both 1-level and 2-level ACDF improve clinical and radiographic outcomes. The choice should be tailored to the patient’s pathology and anatomy while considering the higher dysphagia risk with additional fusion levels. This study highlights the importance of individualized surgical planning to optimize postoperative outcomes while minimizing complications.

## 1. Introduction

Cervical spine disease is a highly prevalent and disabling condition worldwide [1]. While neck pain is often episodic and can be treated conservatively, more severe cases often require surgical intervention. While many patients experience episodic neck pain that responds to conservative treatment, others develop progressive symptoms that require surgical intervention. Cervical spondylotic myelopathy, caused by degenerative narrowing of the spinal canal and compression of the cord, accounts for more than half of non-traumatic spinal cord injuries in North America. Patients may also present with cervical radiculopathy, characterized by nerve root compression leading to arm pain, sensory disturbances, or motor weakness. Together, these conditions represent a major source of morbidity and healthcare utilization [2,3].

Anterior cervical discectomy and fusion (ACDF) is one of the most commonly performed procedures for degenerative cervical pathology. It is indicated in patients with symptomatic myelopathy, radiculopathy, and select cases of trauma or deformity [4,5].

ACDF is highly effective in decompressing neural elements, restoring cervical alignment, and stabilizing the spine. However, surgical planning often requires determining not only whether fusion is necessary, but also how many levels should be fused. Single-level fusion may be appropriate for localized disease, while 2-level fusion is considered when multiple adjacent segments are involved or when greater sagittal alignment correction is required. Although the general technique is similar, extending fusion to an additional level increases operative complexity and may raise the risk of complications such as dysphagia, pseudoarthrosis, or adjacent segment degeneration [6].

Numerous studies have compared 1-level with 2-level ACDF in terms of operative outcomes, radiographic correction, complications, and patient-reported outcome measures. Yet no consensus has emerged regarding superiority [3,7]. Importantly, the choice between 1-level and 2-level procedures is not interchangeable, as surgical indications are patient-specific and depend on the extent of pathology and anatomy. Understanding their relative risks and benefits is therefore critical for guiding individualized surgical decision-making.

Recent projections suggest that the number of ACDF procedures performed in the United States will rise from approximately 153,000 cases in 2020 to over 170,000 cases by 2040, largely due to population aging and the increasing burden of degenerative spine disease. Clarifying the comparative outcomes of 1-level versus 2-level fusion is therefore essential for surgical planning, minimizing complications, and optimizing recovery [8,9].

To address this gap, the present study provides the first systematic review and meta-analysis directly comparing 1-level and 2-level ACDF in terms of operative parameters, postoperative complications, radiographic outcomes, and patient-reported measures. This review was conducted according to PRISMA guidelines. We systematically searched major databases to identify comparative studies of 1-level versus 2-level ACDF and assessed the risk of bias using established tools. Our aim is to synthesize the best available evidence to support clinical decision-making while acknowledging the unique risks associated with multilevel fusion.

## 2. Materials and Methods

### 2.1. Search Strategy

This systematic review and meta-analysis was conducted in accordance with the Preferred Reporting Items for Systematic Reviews and Meta-Analyses (PRISMA) guidelines. The protocol for this review was not registered in PROSPERO or any other registry

Our search was performed in PubMed, Embase, Scopus, and Cochrane Library from inception through 10 July 2024. We used the following keywords: “Single”, “One”, “1”, “Two”, “2”, “Level*”, “ACDF”, and “Anterior Cervical Decompression and Fusion”, “Anterior Cervical Discectomy and Fusion”, and combined them using Boolean operators “AND” and “OR” to identify articles reporting on outcomes of 1-level vs. 2-level ACDF. Additional articles were identified after reviewing the reference lists of the included studies.

### 2.2. Eligibility Criteria

Inclusion criteria consisted of English- and non-English-language studies comparing 1-level with 2-level ACDF. Exclusion criteria consisted of non-comparative studies, review articles, editorial commentaries, case reports, and database studies to avoid patient overlap [1,2].

Two authors independently and in duplicate performed an initial title and abstract screening, followed by full-text screening to identify the articles that satisfied our eligibility criteria, and proceeded with data extraction. A third senior independent author was consulted to discuss and resolve disagreements when present.

### 2.3. Data Extraction

Study characteristics and outcomes from each included article were extracted and tabulated using a Microsoft Excel spreadsheet (Version 2007; Microsoft Corporation, Redmond, WA, USA). Study characteristics included study design, indication for surgery, sample sizes by number of levels fused, and patient demographics such as mean age, sex, mean body mass index (BMI), and mean last follow-up time. Our outcomes of interest included surgery-related outcomes such as operating room time (OR time), estimated blood loss (EBL), and length of stay (LOS); postoperative complications including dysphagia, adjacent segment disease, and revision surgery; radiographic parameters including cervical lordosis (CL), cervical sagittal vertical axis (cSVA), and T1 slope; PROMs including Neck Disability Index (NDI), Visual Analogue Scale for neck (VAS neck) and arm (VAS arm) pain.

### 2.4. Data and Statistical Analysis

Review Manager 5.4 (RevMan, Version 5.4; The Cochrane Collaboration, London, UK) was used for statistical analysis. Weighted means of radiographic parameters and PROMs at final follow-up were calculated for patients in studies reporting them, based on the number of levels fused (1-level vs. 2-level), and compared using independent t-tests. Mean improvements from preoperative to postoperative scores of the extracted radiographic parameters and PROMs were reported as mean differences (MDs). Subgroup analyses were performed to compare outcomes by number of levels fused (1-level vs. 2-level). We used a 95% confidence interval (CI), and a random-effects model was applied when considerable heterogeneity was observed (*p* ≤ 0.05 on the Q test or I^2^ > 50%). The random-effects model accounts for both within-study and between-study variance, allowing for more conservative pooled estimates in the presence of variability among studies. Weighted means were used for continuous variables, while odds ratios were used for categorical variables. Publication bias was not formally assessed because none of the outcomes included 10 or more studies, which is the recommended minimum for meaningful interpretation of funnel plots [10].

### 2.5. Risk of Bias Assessment (RoB)

Two authors independently and in duplicate performed the risk-of-bias assessment for all included studies using the Risk Of Bias In Nonrandomized Studies of Interventions (ROBINS-I) tool to assess the quality of comparative nonrandomized studies. The Cochrane risk-of-bias tool was used in a similar manner to assess the risk of bias for randomized studies. Disagreement was resolved by a third senior author.

## 3. Results

**Figure 1:**
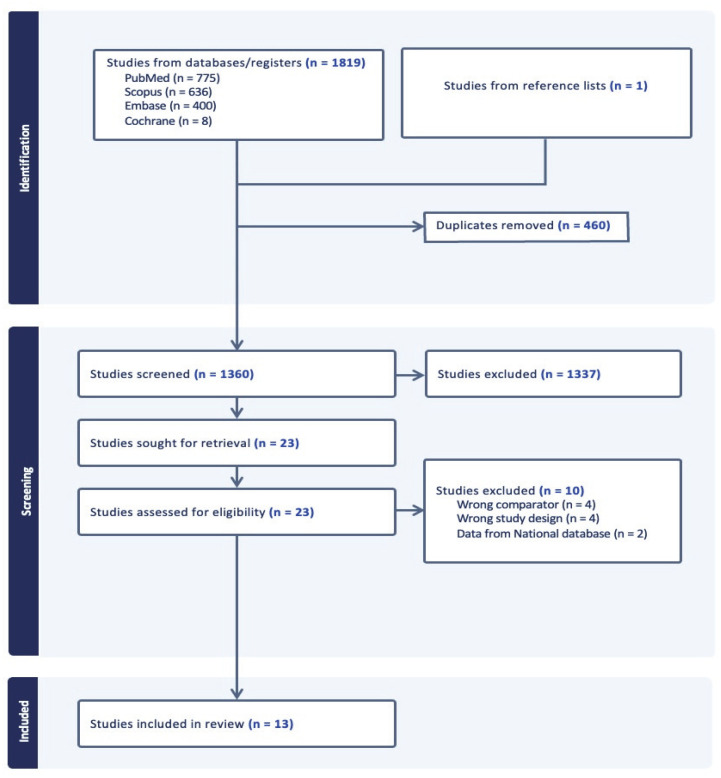
PRISMA flow diagram of the included studies.

Our initial search identified 1819 articles. After the removal of duplicates, 1360 articles proceeded to title and abstract screening. A total of 24 articles reached the full-text screening, and a total of 13 studies met the inclusion criteria and were included in our systematic review and meta-analysis (Figure 1). Of these thirteen studies, one was a randomized controlled trial [3], three were prospective studies [7,11,12], and the remaining nine were retrospective studies [6,13,14,15,16,17,18,19,20]. These studies included 2091 patients, with 1078 patients belonging to the 1-level and 1013 belonging to the 2-level ACDF group. The main characteristics of the included studies are summarized in Table 1.

**Figure 2:**
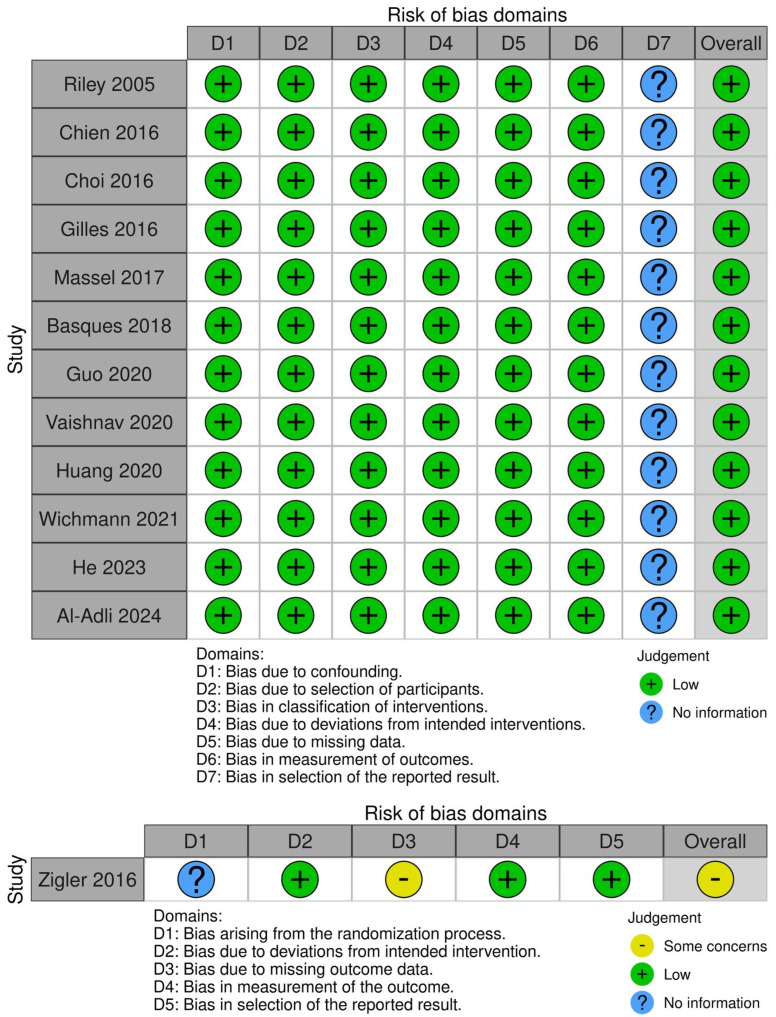
Risk of bias assessment for all included studies [3,6,7,11,12,13,14,15,16,17,18,19,20].

All studies were defined as having low risk of bias, except one assessed as having some concerns [3] (Figure 2). Across the included studies, 1-level and 2-level ACDF were compared within the same patient cohorts under similar surgical techniques and endpoints. Risk-of-bias evaluations (ROBINS-I and Cochrane) showed low concern for confounding, supporting the validity of these comparisons. (Figure 2).

### 3.1. Surgery-Related Outcomes

**Figure A1:**
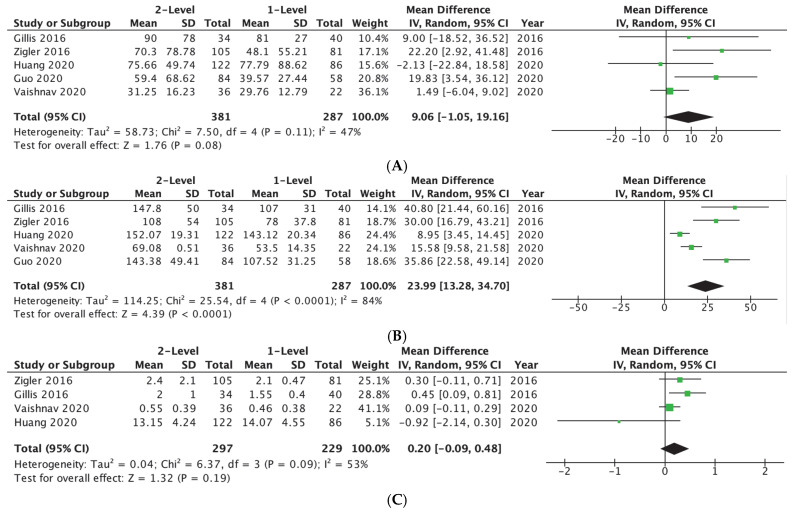
(**A**) Forest plot showing the EBL mean difference between 1-level and 2-level ACDF. (**B**) Forest plot showing the OR time mean difference between 1-level and 2-level ACDF. (**C**) Forest plot showing the LOS mean difference between 1-level and 2-level ACDF [3,11,15,17,18].

Five studies [3,11,15,17,18], including 668 patients, reported EBL and OR time (287 undergoing 1-level ACDF and 381 undergoing 2-level ACDF). No statistically significant difference was seen in EBL (MD = 9.06 [−1.05, 19.16] mL; *p*-value = 0.08) (Appendix A Figure A1A), while 2-level ACDF showed statistically longer OR time (MD = 23.99 [13.28, 34.70] min]; *p*-value < 0.001) (Appendix A Figure A1B). Four studies [3,15,17,18], including 526 patients, reported LOS (221 undergoing 1-level ACDF and 297 undergoing 2-level ACDF) with no statistically significant difference between fusion levels (MD = 0.20 [−0.09, 0.48] days]; *p*-value = 0.19) (Appendix A Figure A1C) (Table 2).

### 3.2. Complications

**Figure A2:**
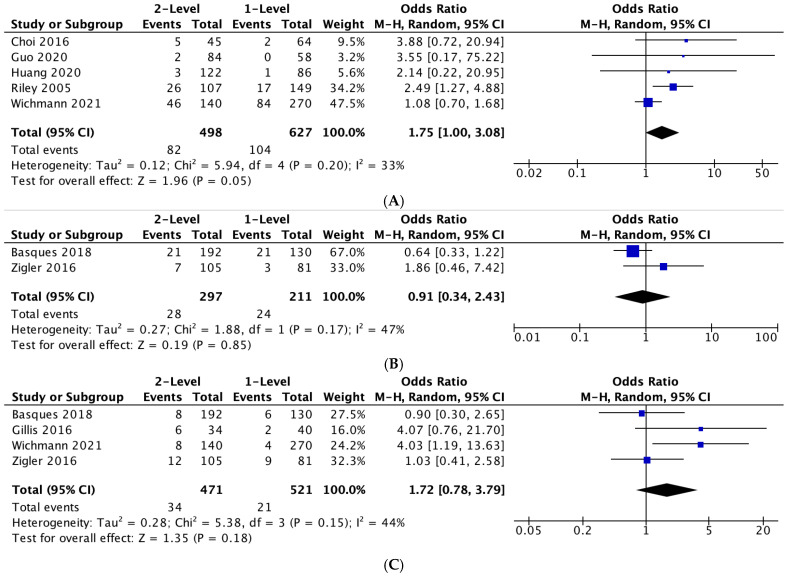
(**A**) Forest plot showing the odds of developing dysphagia between 1-level and 2-level ACDF. (**B**) Forest plot showing the odds of developing adjacent segment disease between 1-level and 2-level ACDF. (**C**) Forest plot showing the odds of undergoing revision surgery between 1-level and 2-level ACDF [3,6,11,13,14,15,18,19].

Five studies [11,13,14,18,19], including 1125 patients, reported on dysphagia (627 undergoing 1-level ACDF and 498 undergoing 2-level ACDF), with 2-level ACDF showing significantly higher odds of developing dysphagia (OR = 1.75 [1.00, 3.08]; *p*-value = 0.05) (Appendix A Figure A2A). Two studies [3,6], including 508 patients, reported data on adjacent segment disease (211 undergoing 1-level ACDF and 297 undergoing 2-level ACDF). No statistically significant difference was seen between fusion levels (OR = 0.91 [0.34, 2.43]; *p*-value = 0.85) (Appendix A Figure A2B). Four studies [3,6,15,19], including 992 patients, reported data on revision surgery (521 undergoing 1-level ACDF and 471 undergoing 2-level ACDF). No statistically significant difference was seen between fusion levels (Appendix A Figure A2C) (Table 2).

### 3.3. Radiographic Parameters

Seven studies [6,7,11,14,15,18,20], including 1022 patients, reported data on CL (460 undergoing 1-level ACDF and 562 undergoing 2-level ACDF). The same four studies [6,11,15,20], including 629 patients, reported data on cSVA and T1S (272 patients undergoing 1-level ACDF and 357 undergoing 2-level ACDF). At final postoperative follow-up, the 1-level ACDF group had mean CL = 13.79 ± 10.63, mean cSVA = 25.91 ± 13.90, and mean T1S = 29.99 ± 8.46, whereas the 2-level ACDF group had mean CL = 13.64 ± 10.65, mean cSVA = 22.96 ± 12.04, and mean T1S = 28.70 ± 8.02, with statistically higher values for 1-level ACDF in terms of T1 Slope and cSVA (*p*-values = 0.05 and 0.005, respectively) (Table 3).

**Figure A3:**
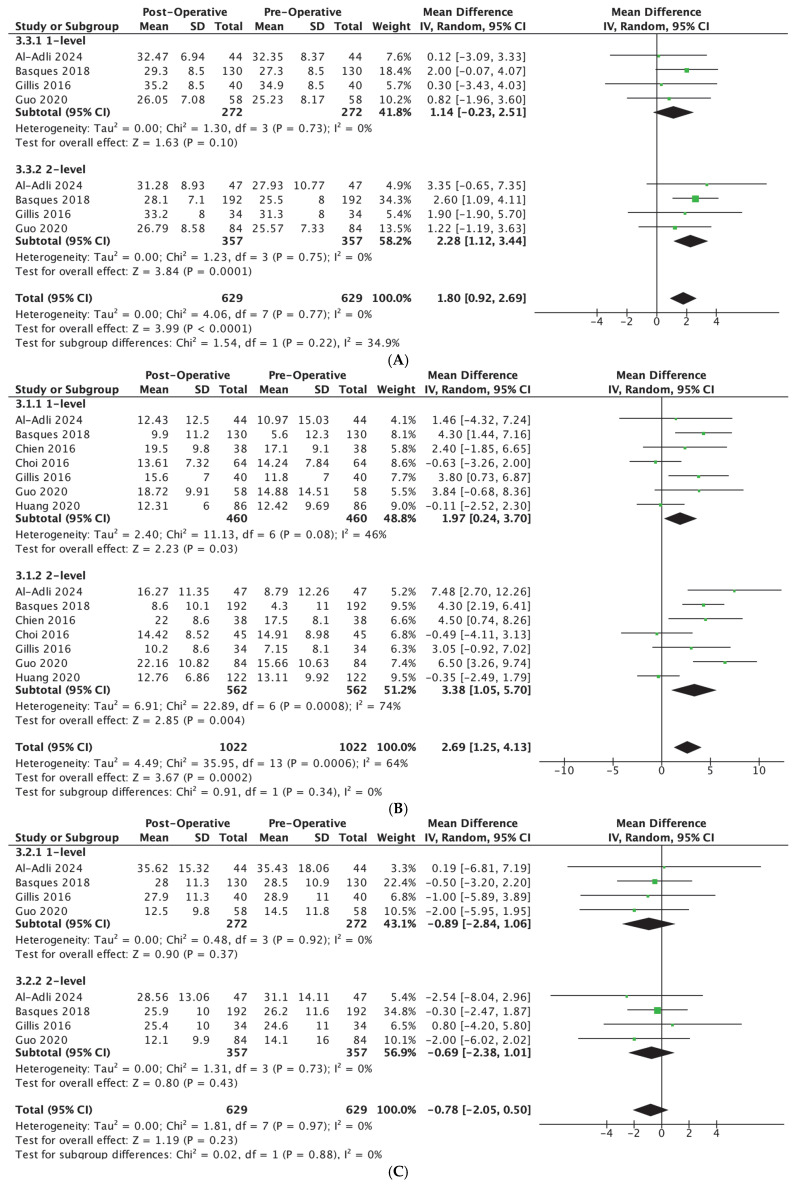
(**A**) Forest plot showing the mean change in T1S from preoperative to postoperative ACDF comparing 1-level to 2-level fusion. (**B**) Forest plot showing the mean change in C2–C7 cervical lordosis from preoperative to postoperative ACDF comparing 1-level to 2-level fusion. (**C**) Forest plot showing the mean change in cSVA from preoperative to postoperative ACDF comparing 1-level to 2-level fusion [6,7,11,14,15,18,20].

In terms of change from preoperative to 1-year follow-up, 1-level ACDF patients failed to maintain significant correction regarding T1S, with a mean difference of 1.14° [95% CI, −0.23, 2.51] (*p*-value = 0.10), while 2-level ACDF patients maintained significant correction, with a mean difference of 2.28° [95% CI, 1.12, 3.44] (*p*-value < 0.01) (Appendix A Figure A3A). Both the 1-level ACDF and the 2-level ACDF patients maintained significant correction for CL, with mean differences of 1.97° [95% CI, 0.24, 3.70] (*p*-value = 0.03) and 3.38° [95% CI, 1.05, 5.70] (*p*-value = 0.004), respectively (Appendix A Figure A3B). Moreover, both the 1-level ACDF and the 2-level ACDF patients did not maintain significant correction for cSVA with mean differences of 0.89 mm [95% CI, −2,84, 1.06] (*p*-value = 0.37) and 0.69 mm [95% CI, −2.38, 1.01] (*p*-value = 0.43) (Appendix A Figure A3C) (Table 4).

### 3.4. Patient-Reported Outcomes

Six studies [6,14,17,19], including 1020 patients, reported VAS arm scores (555 undergoing 1-level ACDF and 465 undergoing 2-level ACDF). Eight studies [6,7,12,14,17,19], including 1166 patients, reported VAS neck scores (637 undergoing 1-level ACDF and 529 undergoing 2-level ACDF). Seven studies [6,7,12,15,17,19], including 1057 patients, reported NDI scores (573 undergoing 1-level ACDF and 484 undergoing 2-level ACDF). At last follow-up, the 1-level ACDF group had mean VAS arm = 1.54 ± 3.07, mean VAS neck = 2.07 ± 2.89, and mean NDI = 19.65 ± 18.89, whereas the 2-level ACDF group had mean VAS arm = 2.21 ± 2.94, mean VAS neck = 2.02 ± 2.64, and mean NDI = 20.8 ± 21.33, with statistically higher VAS arm values for 2-level ACDF (*p*-value = 0.005) (Table 3).

**Figure A4:**
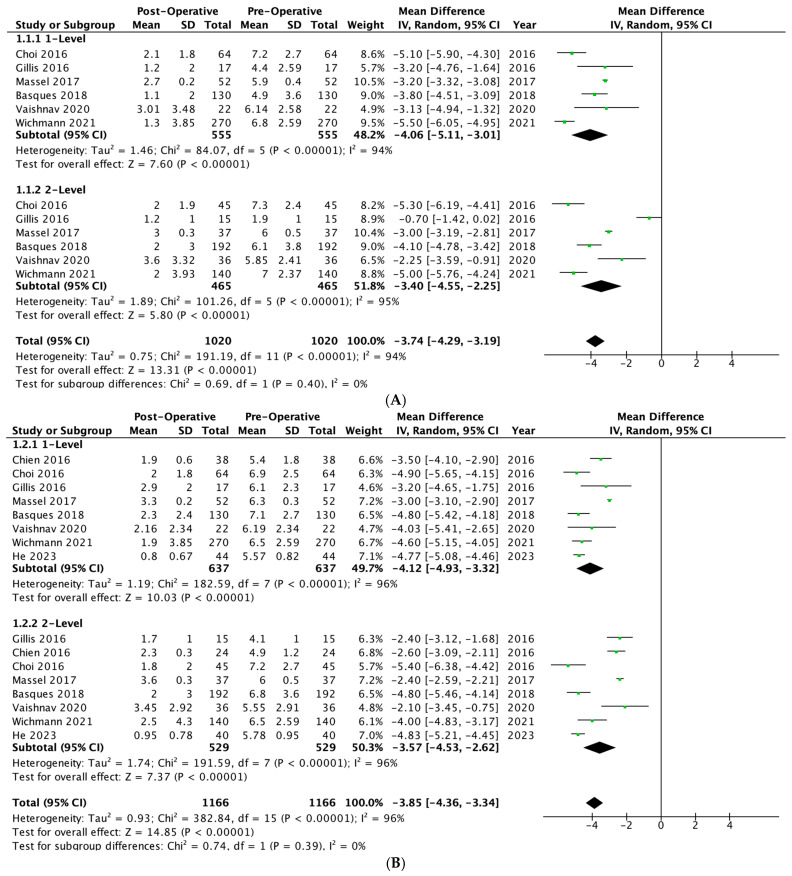

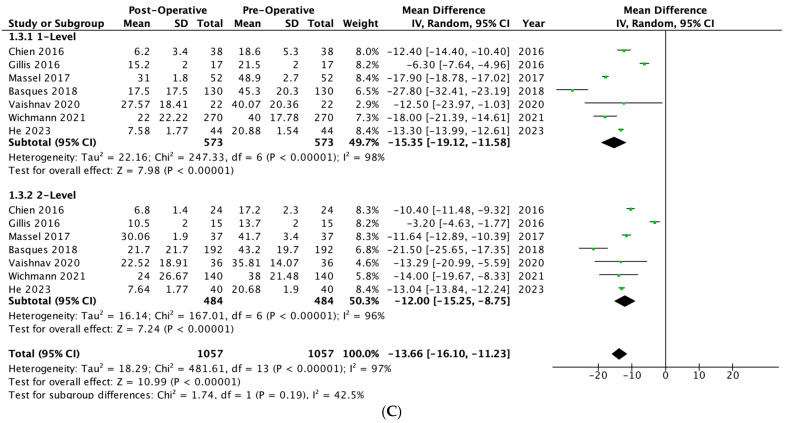
(**A**) Forest plot showing the mean change in VAS arm from preoperative to postoperative ACDF comparing 1-level to 2-level fusion. (**B**) Forest plot showing the mean change in VAS neck from preoperative to postoperative ACDF comparing 1-level to 2-level fusion. (**C**) Forest plot showing the mean change in NDI from preoperative to postoperative ACDF comparing 1-level to 2-level fusion [6,7,12,14,15,16,17,19].

In terms of change from preoperative to 1-year follow-up, 1-level ACDF and 2-level ACDF patients maintained significant improvements in each respective score. VAS arm improved by 4.06 [95% CI, 3.01, 5.11] (*p*-value < 0.01) in the 1-level ACDF group compared with 3.40 [95% CI, 2.25, 4.55] (*p*-value < 0.01) improvement in the 2-level ACDF group (Appendix A Figure A4A). VAS neck improved by 4.12 [95% CI, 3.32, 4.93] (*p*-value < 0.01) in the 1-level ACDF group compared with 3.57 [95% CI, 2.62, 4.53] (*p*-value < 0.01) in the 2-level ACF group (Appendix A Figure A4B). NDI improved by 15.35 [95% CI, 11.58, 19.12] (*p*-value < 0.01) in the 1-level ACDF group compared with 12.00 [95% CI, 11.23, 16.10] (*p*-value < 0.01) in the 2-level ACDF group (Appendix A Figure A4C) (Table 4).

## 4. Discussion

Operating room time is often associated with the complexity of the surgical procedure, with more complicated surgeries necessitating longer time and vice versa [1,21]. In this study, 2-level ACDF was associated with longer operating room times compared to 1-level ACDF. The increased number of fusion levels likely necessitated more tissue dissection and manipulation, which explains the longer procedure time. While this did not translate to increased EBL or longer hospital stays, longer operative times are known to have meaningful implications for both patients and the healthcare system. Longer procedures, for example, increase time under anesthesia, which has been associated with a higher risk of cardiopulmonary complications, particularly in older patients with comorbid conditions who make up a large proportion of patients undergoing ACDF [22,23]. Additionally, longer operative times lead to increased costs for both patients and hospital systems [24].

Moreover, measured postoperative complications were similar between the cohorts, except for 80% higher odds of developing dysphagia in patients undergoing 2-level ACDF compared to 1-level ACDF [25]. This result was borderline significant (*p* = 0.05), and therefore should be interpreted with caution, as it may reflect a trend rather than a definitive difference. This finding aligns with previous reports, which suggest that the risk of dysphagia increases with the number of fusion levels, likely due to the potential for injuring adjacent structures [26]. Beyond patient discomfort, dysphagia can significantly impair oral intake, which can compromise nutrition, wound healing, and overall recovery [27]. While most cases of ACDF-related dysphagia are transient, a subset of patients can have persistent or permanent dysphagia [28]. Patients with mild to moderate dysphagia can benefit from behavioral treatments such as postural changes, sensory enhancements, swallowing maneuvers, and dietary modifications without the need to rely on medications [13,29]. However, for those with severe and chronic dysphagia, smooth muscle relaxants may be beneficial in relieving symptoms after having ruled out other potential causes of dysphagia [30].

In terms of radiographic parameters, patients with 2-level ACDF had greater correction in T1 slope as well as cervical lordosis. Correction of cervical alignment is desirable due to its association with decreased mechanical strain on adjacent segments, mitigating long-term degeneration of the cervical spine [31]. Conversely, inadequate correction of alignment may predispose patients to persistent pain and the need for revision surgery [32]. While greater correction of cervical alignment parameters is desirable, it should be noted that patients initially presenting with worse cervical kyphosis may be more likely to receive an additional level of fusion to correct their cervical deformity. Surgeons should weigh the possibility of greater improvement to cervical alignment against the increased operative time and increased risk for dysphagia associated with 2-level ACDF per our findings. Overall, these findings shed light on the importance of individualizing the number of fusion levels during ACDF based on the patient’s anatomy and disease pathology, to ensure adequate correction of cervical alignment, correlating with improved clinical and functional outcomes [32]. Recent work using 3D posturography in lumbar surgery has further demonstrated the value of advanced methods in evaluating postoperative sagittal parameters and suggests a potential role for such approaches in future cervical spine research [33].

This study further confirmed that both 1-level and 2-level ACDF patients achieved statistically significant improvement in measured VAS arm, VAS neck, and NDI scores. Moreover, both cohorts achieved the minimal clinically important difference (MCID) of 2.6 for VAS neck and 8.65 for NDI [34]. However, neither cohort achieved the MCID for VAS arm of 4.1, although 1-level patients nearly reached this threshold with an average improvement of 4.06 [34]. This indicates that while both groups achieved clinically meaningful gains in VAS neck and NDI, recovery of arm-related symptoms may be less robust after 2-level ACDF, possibly reflecting greater baseline nerve involvement and slower biological recovery. These results highlight that statistical significance does not always translate to a clinically meaningful benefit across all outcome domains, and MCID thresholds provide important context when interpreting PROMs. Liu et al. suggested that achieving MCID in VAS neck and NDI but not in VAS arm may be explained by the fact that improvements in neck-related pain measures can be quickly achieved postoperatively through additional interventions such as physical therapy. In contrast, arm-related pain, which is determined by the healing of the compressed nerve, tends to be a slower and more biologically constrained process [34].

While patients in both cohorts presented with generally similar preoperative PROM scores, patients requiring 2-level ACDF likely had worse radiographic and clinical presentations that necessitated the additional level of fusion [6,11]. It is possible that 2-level ACDF patients initially sustained more pronounced nerve injury, contributing to differences in VAS arm scores and being further from achieving MCID compared to 1-level ACDF patients. Overall, patients in both cohorts achieved clinically and statistically significant improvements in PROMs, emphasizing the benefits of ACDF regardless of the number of levels fused.

In summary, our findings highlight the benefits of undergoing ACDF surgery in achieving adequate alignment correction, which translates into significant improvements in PROMs irrespective of the number of levels fused, and highlight the need to tailor surgical treatment to each individual’s baseline pathology and anatomy while considering the higher odds of developing dysphagia with increasing levels of fusion.

Our findings should be interpreted in the context that 1-level and 2-level ACDF are not interchangeable procedures, as the indication for surgery is determined by individual patient pathology and anatomy. Surgical decision-making therefore requires consideration of clinical presentation, radiographic deformity, and the balance between potential alignment correction and risk of complications. This aligns with recent perspectives emphasizing individualized treatment strategies in cervical spine surgery [35].

## 5. Strengths and Limitations

Our study is the first systematic review and meta-analysis to compare 1-level with 2-level ACDF in terms of surgery-related outcomes, postoperative complications, radiographic parameters, and PROMs. Nevertheless, it is not without limitations. First, nine of the thirteen included studies were retrospective in design, which inherently introduces more bias compared to prospective studies, particularly regarding patient selection and retention. Second, the included studies assessed different indications for surgery, which could affect the generalizability of our findings. Third, due to heterogeneity in reported follow-up times, we cannot determine how comparisons between cohorts change over time or whether the observed differences regarding dysphagia and VAS arm persist as patients continue to heal. Taken together, these sources of heterogeneity in study design, surgical indications, and follow-up duration limit the reliability and generalizability of our pooled conclusions. Retrospective designs increase the risk of selection bias, varied surgical indications introduce differences in baseline disease severity, and inconsistent follow-up times may underestimate or overestimate complications such as adjacent segment disease. Therefore, our findings should be interpreted with caution. Fourth, there is significant heterogeneity in how dysphagia is defined across the literature, which contributes to variability in reported rates. Moreover, the included studies did not specify whether dysphagia was transient or persistent, or whether it required intervention, making it unclear whether this difference is clinically significant. Fifth, although our analysis focused on dysphagia, adjacent segment disease, and revision surgery as the main adverse outcomes, other complications were rarely or inconsistently reported and therefore could not be reliably pooled. Sixth, the included studies lacked data on motion changes, which are important when assessing adjacent segment disease. Since increased fusion levels can alter cervical biomechanics and accelerate adjacent segment degeneration, future studies with dynamic imaging and long-term follow-up are needed to better assess these considerations. Finally, our protocol was not prospectively registered in a database such as PROSPERO, which may introduce reporting bias and should be considered a limitation. Overall, while this review synthesized the principal reported endpoints, detailed guidance for clinical decision-making will require prospective, standardized investigations and dedicated guideline development.

## 6. Conclusions

This systematic review and meta-analysis demonstrates that both 1-level and 2-level ACDF are effective in achieving significant improvements in radiographic parameters and PROMs. While 2-level ACDF was associated with a longer procedure, this did not result in more blood loss or longer hospital stays compared to 1-level ACDF. Moreover, patients undergoing 2-level ACDF showed higher odds of developing dysphagia, although this association was borderline significant and should be interpreted with caution. This emphasizes the need to carefully consider the number of fusion levels, particularly in patients with predisposing risk factors for dysphagia. Finally, the improvements in alignment that were shown with 2-level ACDF suggest that this procedure may be particularly beneficial for patients with more severe presenting deformities. Future research should focus on prospective studies with longer follow-up, standardized reporting of complications, and direct comparisons of 2-level versus multilevel (≥3) ACDF, as well as alternative motion-preserving strategies, to better guide surgical decision-making.

## Figures and Tables

**Table 1:** Overview of the included studies.

Author Year	Study Design	Indication for Surgery	Number of Levels Fused	Number of Participants	Mean Age ± SD (Years)	Sex	Mean BMI ± SD (kg/m^2^)	Last Follow-Up Time Point (Months)
Riley 2005 [13]	Retrospective Study	Myelopathy or Radiculopathy	1-level	149	48.2 ± 10.74	134 M, 122 F	NR	24
			2-level	107				
Chien 2016 [7]	Prospective Study	Myelopathy or Radiculopathy	1-level	38	53.4 ± 12.0	21 M, 17 F	NR	12
			2-level	24	56.3 ± 10.4	15 M, 9 F	NR	
Zigler 2016 [3]	Randomized controlled trial	Myelopathy or Radiculopathy	1-level	81	44.0 ± 8.21	36 M, 45 F	27.4 ± 4.18	24
			2-level	105	46.2 ± 7.99	45 M, 60 F	28.1 ± 4.19	
Choi 2016 [14]	Retrospective Study	Spondylotic Myelopathy	1-level	64	52.2 ± 14.0	44 M, 20 F	NR	12
			2-level	45	54.2 ± 17.1	27 M, 18 F	NR	
Gillis 2016 [15]	Retrospective Study	Myelopathy or Radiculopathy or Spondylosis	1-level	40	52.0 ± 9.33	37 M, 37 F	27.1 ± 1	12
			2-level	34				
Massel 2017 [16]	Retrospective Study	Spondylosis or Spondylolisthesis or Herniated Nucleus Pulposus	1-level	52	49.7 ± 8.8	50 M, 49 F	NR	12
			2-level	37				
Basques 2018 [6]	Retrospective Study	Myelopathy or Radiculopathy	1-level	130	46.6 ± 11.9	61 M, 69 F	28.4 ± 6.0	28
			2-level	192	50.2 ± 10.0	97 M, 95 F	28.2 ± 6.1	
Guo 2020 [11]	Prospective study	Myelopathy	1-level	58	51.93 ± 8.90	33 M, 25 F	24.80 ± 3.06	12
			2-level	84	57.05 ± 9.89	47 M, 37 F	24.54 ± 3.29	
Vaishnav 2020 [17]	Retrospective Study	Myelopathy or Radiculopathy	1-level	22	48.06 ± 10.68	13 M, 9 F	29.13 ± 5.18	3
			2-level	36	54.17 ± 8.67	14 M, 2 F	28.65 ± 6.29	
Huang 2020 [18]	Retrospective Study	Myelopathy or Radiculopathy	1-level	86	51.47 ± 11.46	52 M, 34 F	24.16 ± 2.88	18 ± 3.46
			2-level	122	52.39 ± 8.68	70 M, 52 F	23.82 ± 3.31	
Wichmann 2021 [19]	Retrospective Study	Radiculopathy	1-level	270	52 ± 10	124 M, 146 F	NR	12
			2-level	140	55 ± 9.8	67 M, 73 F		
He 2023 [12]	Prospective Study	Spondylosis	1-level	44	52.79 ± 5.55	26 M, 18 F	23.19 ± 2.49	12
			2-level	40	54 ± 5.94	22 M, 18 F	22.08 ± 2.70	
Al-Adli 2024 [20]	Retrospective Study	Myelopathy or Radiculopathy	1-level	44	59.9 ± 9.1	18 M, 73 F	30.5 ± 5.7	2.6 ± 0.96
			2-level	47				

**Table 2:** Summary of the surgery-related outcomes and odds ratios of the complications between 2-level and 1-level fusion ACDF.

Surgery-Related Outcomes	Mean Difference [95% CI]	*p*-Value
EBL	9.06 [−1.05, 19.16] mL	0.08
OR time	23.99 [13.28, 34.70] min	**<0.001**
LOS	0.20 [−0.09, 0.48] days	0.19
Complications	Odds Ratio [95% CI]	*p*-value
Dysphagia	1.75 [1.00, 3.08]	**0.05**
Adjacent segment disease	0.91 [0.34, 2.43]	0.85
Revision surgery	1.72 [0.78, 3.79]	0.18

EBL, estimated blood loss; OR, operating room; LOS, length of hospital stay; CI, confidence interval. *p* < 0.05 is considered significant and is shown in bold.

**Table 3:** Summary of the mean postoperative radiographic parameters and patient-reported outcomes.

Mean Postoperative Scores (Mean ± SD)			
Radiographic Parameters	1-Level	2-Level	*p*-value
cSVA	25.91 ± 13.90	22.96 ± 12.04	**0.005**
Cervical lordosis	13.79 ± 10.63	13.64 ± 10.65	0.83
Patient-reported outcomes	1-Level	2-Level	*p*-value
VAS arm	1.54 ± 3.07	2.11 ± 3.3	**0.005**
VAS neck	2.07 ± 2.89	2.16 ± 3.22	0.59
NDI	19.65 ± 18.89	20.8 ± 21.33	0.35

cSVA, cervical sagittal vertical axis; NDI, Neck Disability Index; VAS, Visual Analogue Scale; SD, standard deviation. *p* < 0.05 is considered significant and is shown in bold.

**Table 4:** Summary of the mean change scores from preoperative to 1-year follow-up in radiographic parameters and patient-reported outcomes.

	1-Level		2-Level	
Radiographic Parameters	Mean Difference [95% CI]	*p*-Value	Mean Difference [95% CI]	*p*-Value
Cervical lordosis	1.97 [0.24, 3.70]	0.03	3.38 [1.05, 5.70]	**<0.01**
cSVA	−0.89 [−2.84, 1.06]	0.37	−0.69 [−2.38, 1.01]	0.43
Patient-reported outcomes	Mean Difference [95% CI]	*p*-value	Mean Difference [95% CI]	*p*-value
VAS arm	−4.06 [−5.11, −3.01]	<0.01	−3.40 [−4.55, −2.25]	**<0.01**
VAS neck	−4.12 [−4.93, −3.32]	<0.01	−3.57 [−4.53, −2.62]	**<0.01**
NDI	−15.35 [−19.12, −11.58]	<0.01	−12.00 [−16.10, −11.23]	**<0.01**

cSVA, cervical sagittal vertical axis; NDI, Neck Disability Index; VAS, Visual Analogue Scale; CI, confidence interval. *p* < 0.05 is considered significant and is shown in bold.

## Data Availability

All data used in this study were obtained from previously published articles that are publicly available.
